# Data-driven ELNES/XANES analysis: predicting spectra, unveiling structures and quantifying properties

**DOI:** 10.1093/jmicro/dfaf038

**Published:** 2025-09-11

**Authors:** Teruyasu Mizoguchi

**Affiliations:** Institute of Industrial Science, The University of Tokyo, 4-6-1 Komaba, Meguro, Tokyo 113-8505, Japan

**Keywords:** EELS, XAFS, machine learning, data driven, ELNES, XANES

## Abstract

Core-loss spectroscopies using electrons and X-rays, such as electron energy loss near-edge structures (ELNES) and X-ray absorption near-edge structures (XANES), are indispensable tools for materials characterization and development. These techniques provide detailed insights into atomic environments, chemical bonding, and vibrational properties that underpin material functionality. Traditionally, ELNES/XANES analyses have relied on qualitative interpretation or comparisons with reference spectra obtained from experiments and/or simulations. Recent advances in data-driven approaches, however, have enabled more quantitative and predictive use of these spectra. This review highlights newly developed data-driven methodologies that extend far beyond conventional ELNES/XANES analysis. These approaches accelerate ELNES/XANES simulations, enable the extraction of radial distribution functions, and quantify multiple material properties directly from spectral data. To enhance the interpretability of machine learning (ML) predictions, sensitivity analysis is employed to elucidate the relationships between specific spectral features and target properties. The rapid growth of open materials databases, coupled with increasingly powerful ML models, has further fueled these developments. Together, these advances would point to a future in which automated, interpretable and scalable spectroscopy serves as a central driver for deeper understandings and accelerated materials discovery.

## Teaser

Our approaches presented in this review have the potential to accelerate ELNES/XANES simulations, enable the extraction of radial distribution functions, and quantify multiple material properties directly from the ELNES/XANES data.

## Introduction

Electron energy loss spectroscopy (EELS), observed using a transmission electron microscope (TEM) or scanning TEM (STEM), is a spectroscopy technique capable of observing zero-loss, low-loss and core-loss spectra. The core-loss region, known as the energy loss near-edge structures (ELNES), is basically equivalent to the X-ray absorption near-edge structures (XANES) obtained using a synchrotron. Both ELNES and XANES originate from electronic transitions from a core orbital to the conduction bands [1, 2]. Since their transitions obey the dipole selection rule, the resulting spectral features reflect the unoccupied partial density of states (PDOS). Accordingly, ELNES and XANES provide insight into the local environments and electronic structures of specific elements within the illuminated region.

Thus, ELNES and XANES have been widely utilized as complementary spectroscopic techniques to investigate the local environments (coordination, bond length, strain, defect and so on) and electronic structure (valence state, ionicity, covalency, charge transfer and so on) of functional oxides [3–10], energy materials [11–16], ceramics and alloys [17–24], two-dimensional materials [25–28] and organic compounds [29–33]. The expansion of these applications has been closely tied to advancements in their experimental equipment. In recent years, significant technological innovations have been reported in both electron microscopy and synchrotron radiation facilities.

In the case of EELS, improvements in monochromator performance have enabled energy resolutions down to a few millielectronvolts. This high-energy resolution allows the extraction of infrared region information that appears near the zero-loss peak [34–40]. Moreover, by leveraging the ­inherently high spatial resolution of transmission electron microscopy, high-spatial-resolution vibrational spectroscopy has also been demonstrated [41–46]. In addition to the developments on the low-energy side, recent efforts have focused on extending the capabilities of EELS toward higher energy-loss regions. Due to limitations such as chromatic aberration and signal intensity, routine acquisition of high-energy EELS data have traditionally been challenging. While early studies reported on such high-­energy measurements [47], recent technological advances, such as the use of constant-power lenses, have facilitated more accessible high-energy EELS, exemplified by the successful detection of the Sb K-edge at around 30 keV [48, 49].

Similarly, in the field of XANES, their instrumental developments have enabled higher energy resolution. For instance, fluorescence-based techniques have mitigated the resolution degradation caused by core-hole lifetime broadening, achieving up to several times improvements in the energy resolution [50–52]. Furthermore, synchrotron radiation offers flexible experimental layouts, enabling operando analyses under conditions that are difficult to realize with electron microscopes. Such operando observations have been successfully conducted in studies, allowing for *in situ* measurements under realistic environmental and reaction conditions [53–55].

Along with these advancements in instrumentation, the experimentally observed ELNES/XANES spectra have become increasingly enriched in both variety and quality. In parallel with these instrumental developments, advancements in the theoretical simulation of ELNES/XANES have also been made for interpreting the experimental spectra. A key issue of the ELNES/XANES simulation is the appropriate treatment of the core hole, which is present in the core orbital at the electronic excitation process [56, 57]. Depending on how the core state and the excited electron are modeled, simulation approaches can be categorized into three types: the one-particle method, such as density functional theory (DFT) with generalized gradient approximation (GGA) [58–64]; the two-particle method, such as the method based on Bethe–Salpeter Equation [65–69]; and the multi-particle method, such as the method using configurational interaction method [70–76]. The theoretical background and applications of these approaches have been reviewed in the other literature by the present authors [77–81].

Beyond spectral reproduction, theoretical tools have also been developed to clarify the correlations between spectral features and underlying material properties [17, 82–84]. By integrating experimental data with theoretical simulations, researchers can extract insights into valence states [85, 86], local atomic coordination environments [87–89], vibrations in solids, liquids and gases [81, 90–92], weak interactions such as van der Waals forces [81, 93] and even local thermal properties of materials [94, 95].

Given these advancements in the experimental and theoretical viewpoints described above, a wide range of novel analytical methods have been proposed. In parallel, data-driven techniques have recently garnered considerable attention in the field of materials science for their ability to extract meaningful insights from large-scale datasets. In particular, machine learning (ML), a core component of artificial intelligence (AI), has been actively applied to materials discoveries, materials simulations and even materials characterizations.

The approaches with ML have enabled the efficient identification of target structures with minimal computational cost, the discovery of high-performance materials and the extraction of information that could not be obtained through conventional experiments or simulations. For instance, data-driven approaches have been effectively utilized for materials discoveries [96–103]. ML has also been used for investigating the structures and properties of lattice defects [104–115], and some ML architectures have also been constructed for the predictions of properties and chemical bonding of materials [116–123].

Applications of ML to experimental data have also become widespread. In the field of electron microscopy, ML has been used to analyze images, spectra and diffraction patterns, including four-dimensional STEM datasets [124–136]. In the XAFS analysis, ML has also been utilized to predict coordination numbers and local atomic environments [137–142]. The authors’ group has also contributed to this field for developing methods for spectral prediction and interpretation using dendrogram-based approaches [143, 144] and by utilizing ELNES/XANES spectra as input to predict local structures, chemical bonding and materials properties [145–155].

The growing use of ML in materials science is strongly correlated to the rapid progress of open science. A research environment that encourages the open sharing of data and code is becoming increasingly established. Comprehensive materials databases such as the Materials Project [156] and NOMAD [157] provide extensive datasets, which have accelerated the development of ML methods and data utilization on a global scale. For instance, widely used pre-trained universal ML interatomic potentials have been constructed using the massive public datasets of atomic energies and forces [158–161]. It is noteworthy that such datasets dedicated for materials developments have been released by big IT companies, such as Microsoft and Google, reflecting the high level of interest in this area [96, 99]. In addition to online repositories, data are also being published in scientific journals dedicated to open data, such as *Scientific Data* and *Data in Brief*.

In the modern science and technology, the ability to access, generate and utilize data, as well as to extract valuable insights from data, is becoming a fundamental factor for determining the research capability.

Building upon the backgrounds described above, this review summarizes recent results from the authors’ group regarding the data-driven approaches for the ELNES/XANES spectra. The author previously published a related review article in 2020 in this journal concerning the dendrogram-based approaches for the spectrum prediction and interpretation and property prediction from the spectrum [146]. The present review focuses on the part of the research outcomes obtained recently [148–151].

**Fig. 1.**
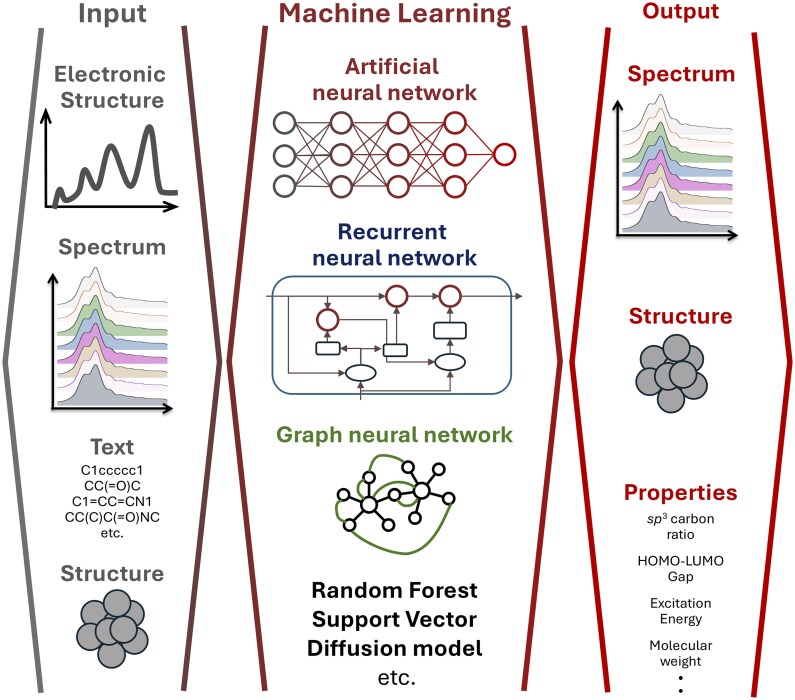
Schematic illustration representing the data-driven approach introducing this review. The various type of input (left) is applied to the suitable machine learning architecture (middle) to obtain the target output (right).

A schematic illustration of this review is shown in Fig. 1. In ML applications to the ELNES/XANES, various types of inputs and training architectures are utilized to obtain diverse outputs. The selection of inputs, the choice of ML architectures, and the resulting outputs vary depending on the objective, data availability, and the expertise. In this review, partial density of states (PDOS), ELNES/XANES, and text were used as the inputs (left side of Fig. 1), and ELNES/XANES, radial distribution functions (RDF), and 12 materials properties were outputs for the predictions (right side of Fig. 1).

I would first introduce approaches for accelerating theoretical calculations of ELNES/XANES. These approaches utilize easily calculatable input data, such as PDOS at the ground state [148]. I also show the methods for predicting ELNES/XANES directly from a text, such as SMILES (simplified molecular input line entry system) strings, without the atomic positions [151]. Next, I would introduce the prediction of local coordination, namely RDF from ELNES/XANES [149]. Furthermore, multiple material properties, in this case 12 properties including excitation energy, HOMO (highest occupied molecular orbital)–LUMO (lowest unoccupied molecular orbital) gap, and molecular weight, were predicted using the ELNES/XANES as input [150]. Finally, I also introduce sensitivity analysis to elucidate how the ML models make their predictions, showing one possible route to overcoming the ‘black box’ criticism frequently directed at ML-based models. This analysis provides insights into the underlying reasoning and enhances the interpretability of data-driven spectroscopic approaches.

## Methodology

### Spectrum database construction

To enable data-driven approaches, the construction of a comprehensive spectral database is essential. In our work, all ELNES/XANES spectra were basically calculated using the CASTEP code [162, 163], which is based on a first-principles plane-wave pseudopotential method. The exchange–correlation functional was approximated using the Perdew–Burke–Ernzerhof (PBE) GGA, and the plane-wave cutoff energy was set to 500 eV. Core-hole effects were introduced by generating and applying an excited pseudopotential to the targeted oxygen atom within a supercell. To minimize interactions among excited atoms under periodic boundary conditions, large super cells with dimensions >10 Å were used. Theoretical transition energies were also calculated following the procedure outlined in the previous report [163]. Further details of the calculated spectra are provided in the corresponding sections.

### ML techniques

When spectral features were used as inputs, a feedforward neural network (FNN) was primarily employed for the predictions. FNNs, being the simplest type of artificial neural network, are well suited to handle high-dimensional inputs such as spectra and capture complex nonlinear correlations among features. The network was trained using the backpropagation algorithm with the Adam optimizer [164], minimizing the mean absolute error between the predicted and target values. Rectified linear units (ReLU) were used as activation functions, and dropout with a rate of 0.5 was applied to hidden layers, except the output layer, to prevent overfitting. Hyperparameters, including the number of hidden layers and regularization parameters, were tuned via 5-fold cross-validation on the validation datasets.

This architecture is particularly effective for predicting ELNES/XANES spectra due to its flexibility in managing multidimensional input–output mappings and capturing intricate dependencies. The input layer receives features computed at low cost (e.g. ground-state electronic structures), while the output layer is the ELNES/XANES.

For instance, the PDOS of the ground state will be used as the input, and the intensity values of ELNES/XANES will be used as the output. This is motivated by Fermi’s golden rule, which states that core-electron excitation is governed by the electronic structure of the ground state. While PDOS can be computed within seconds or minutes using a primitive cell, ELNES/XANES simulations, requiring both ground and excited states within a large supercell, can take hours to days. Detailed preprocessing procedures for the input data are described in each section.

In addition to the PDOS, a sequence-based model for the ELNES/XANES prediction directly from text, such as SMILES, is also introduced. For this task, a recurrent neural network (RNN) was used. The model comprises an input layer, a hidden layer, and an output layer. To process SMILES strings, we converted the canonical SMILES into a one-hot encoded format, resulting in a 100 × 24 matrix [151]. The first dimension (length 100) represents the maximum SMILES string length considered, accommodating large molecules and serving as the input node count. The second dimension (length 24) denotes the set of distinct characters in the SMILES vocabulary.

## Results and discussion

### Acceleration of ELNES/XANES simulation: prediction of ELNES/XANES from ground-state electronic structure

ELNES and XANES are powerful techniques for probing local atomic and electronic structures. While combining experimental spectra with simulations enhances interpretability, such ELNES/XANES simulations are computationally demanding because they require consideration of both ground and excited states. If the excited-state features could instead be reliably predicted from ground-state information, both the efficiency of spectral interpretation and our fundamental understanding of excitation mechanisms could be significantly improved. For this object, we employed an FNN to predict excited-state spectra from ground-state electronic structures.

To train and evaluate the model, we constructed a simulation-based spectrum dataset, which is free from experimental noise and errors. We focused on the oxygen K-edge (O–K edge) spectra of silicon oxide polymorphs because of the following reasons: (i) silicon oxide exhibits a wide variety of polymorphs, enabling the construction of a large and diverse dataset; (ii) the O–K edge can be accurately reproduced using a relatively simple one-particle approach within the framework of the GGA of DFT; and (iii) silicon oxides are technologically important materials used in a broad range of applications, including optics, catalysis and energy storage.

We extracted 188 crystalline silicon oxide structures from the Materials Project database [156] and calculated a total of 1171 O–K edge spectra corresponding to their multiple nonequivalent oxygen sites. To further investigate the effect of structural disorder, we also simulated the O–K edge spectra of amorphous silicon dioxide. Amorphous structures containing 72 silicon and 144 oxygen atoms were generated using classical molecular dynamics simulations [148].

**Fig. 2.**
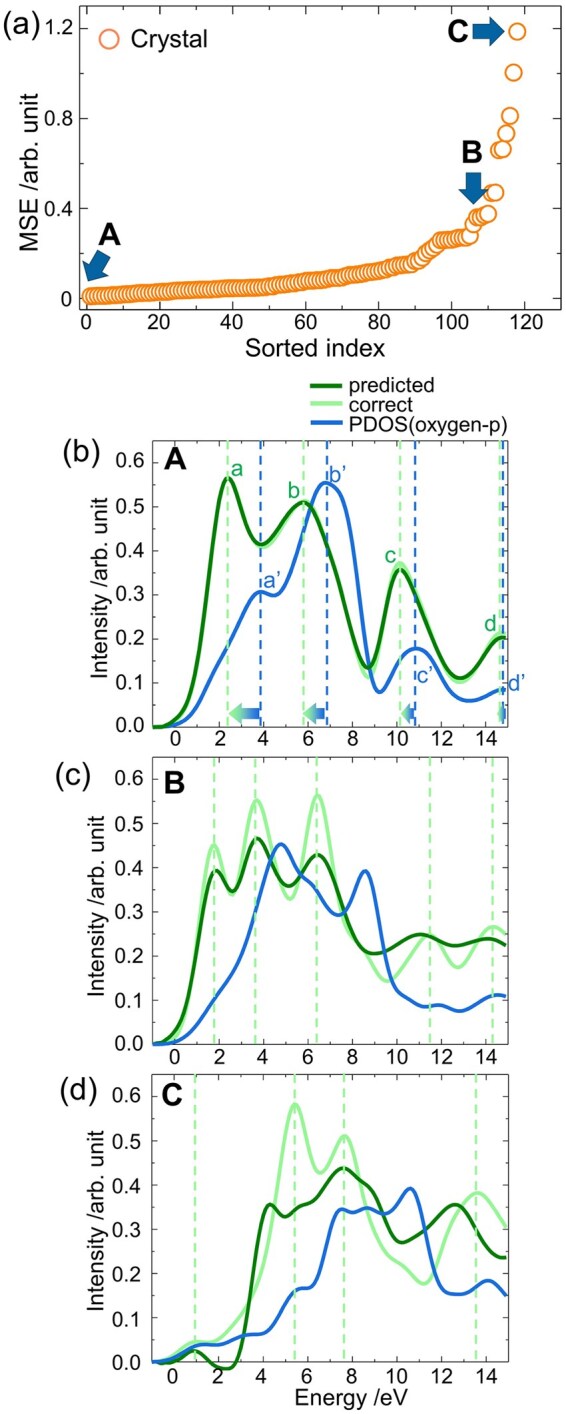
Predictions of the ELNES/XANES with ground state PDOS. (a) Sorted MSEs are scattered by orange symbols. A, B and C, indicated by blue arrows, correspond to the A, B and C data in (b–d). Deep and light green lines are the predicted and accurate spectra, respectively. Blue lines are PDOS in ground states. This figure is adapted from reference [148].

We evaluated the performance of our FNN-based predictive model by calculating the mean squared error (MSE) on the test dataset. Figure 2a presents the distribution of MSE values, where the *x*-axis corresponds to the index of the test data and the y-axis shows the MSEs sorted in ascending order. Over 90% of the test data exhibited MSEs below 0.4, indicating that the FNN could accurately reproduce the ELNES/XANES spectra based on the ground-state PDOS. Importantly, our model requires only the ground-state PDOS of the primitive cell as input, allowing it to predict spectra with computational costs that are orders of magnitude, approximately several thousand times, lower than those of conventional first-principles ELNES/XANES simulations.

To further assess the quality of the predictions, three spectra, labeled A, B and C in Fig. 2a, are shown in Fig. 2b–d. In each figure, the predicted spectrum is shown in green, and the reference (simulated) spectrum is shown in light green. Spectrum A (MSE = 0.01) shows excellent agreement with the reference, including both peak positions and intensities (Fig. 2b). Spectrum B (MSE = 0.38) still reproduces the major spectral features, such as the relative peak positions (Fig. 2c), although minor discrepancies are visible. By contrast, Spectrum C (MSE = 1.2) fails to accurately reproduce the peak positions and intensities (Fig. 2d).

We further examined the physical basis for the model’s accuracy by analyzing Spectrum A in more detail. In Fig. 2b, peaks in the ELNES/XANES spectrum are labeled a–d, and the corresponding peaks in the PDOS are denoted a′–d′. As can be seen, the peaks in the PDOS are systematically shifted to lower energies in the predicted spectrum, as indicated by the leftward arrows. The mechanisms behind the peak shifts to lower energy can be ascribed to the influence of the core hole. In the present case, the O–K edge has a core hole in its O 1 *s* orbital. The core hole generates a strong Coulomb interaction from the nucleus, and then the wave functions are largely localized near the excited atom; this large Coulomb interaction shifts the peaks to lower energy, i.e. from a′–d′ to a–d in Fig. 2b [56, 57]. Furthermore, the magnitude of the shift is typically larger for lower energy peaks due to the stronger Coulomb interactions from the nucleus near the conduction band edge. Our model successfully captures such a trend, as reflected in the varying lengths of each left-faced arrow in Fig. 2b. These results indicate that the FNN effectively learned the underlying physics associated with the core hole, despite being trained only on ground-state features.

**Fig. 3.**
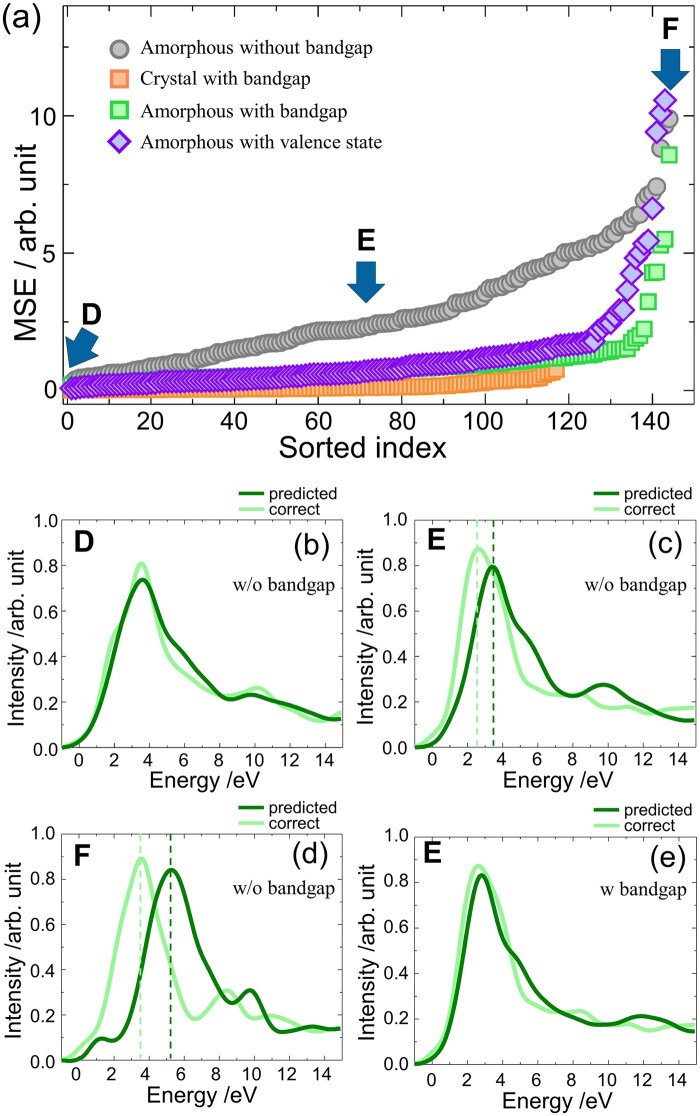
(a) Sorted MSEs are plotted by colored symbols. Gray circles are those of the amorphous without band gap as inputs; orange and green squares are those of the crystalline and amorphous materials considering the band gap, respectively; purple rhombuses denote the MSEs of the amorphous materials considering valence states. (b–e) ELNES/XANES spectra and PDOS for points of D–F. This figure is adapted from reference [148].

We have next applied the trained model to new data. Once a ML model is trained on a given dataset, it can be used to make predictions on different but related datasets. In the first case, we investigated the transferability of our FNN trained on crystalline silicon oxides by applying it to amorphous silicon oxides. Figure 3(a) shows the MSEs obtained for the amorphous structures, and three spectra in Fig. 3b–d correspond to the spectra with the accuracies of D–F, denoted by arrows in Fig. 3a. First, the gray symbols in Fig. 3a are significantly larger, which is roughly one to two orders of magnitude than those observed in the crystalline cases (Fig. 2a), indicating that the present prediction model cannot be used to predict the spectrum of the amorphous structures.

The predicted spectrum shown in Fig. 3b (green line), which had the best MSE at point D, was relatively similar to the correct spectrum (light green line), but the main peaks of the predicted spectra in Fig. 3c and d, which correspond to the points E and F, were noticeably underestimated compared to the reference. These trends suggest that the magnitude of the core-hole effect predicted by the model trained on the crystalline silicon oxides was systematically weaker for the amorphous silicon oxides. In other words, the amorphous silicon oxides exhibit stronger core-hole effects than their crystalline silicon oxides. It is noteworthy that this finding cannot be obtained only by a conventional ELNES/XANES simulation, because the excited state was directly and automatically calculated by the simulation, and the excited state of the crystalline material cannot be ‘transferred’ to the amorphous material.

To address this underestimation, we consider the shielding effects, which involve the core-hole effects. Indeed, the core hole is commonly derived from ‘one’ electronic hole at the core state, so the strength of the Coulomb interactions from the nucleus should be similar among these O–K edges, but we could speculate that the changes in the core-hole effects can be ascribed to the different shielding effect. To implement the shielding effect in the prediction model, the band gap values were used as additional input because of the band structure. We obtained the band gap values with the GGA-PBE simulations at the ground state. We re-trained the FNN using the crystalline dataset, now including band gap information as an additional input, and used it to predict the excited states of both crystalline and amorphous materials. The resulting MSEs with consideration of the band gap are also summarized in Fig. 3a; the orange and green symbols represent the MSEs for crystalline and amorphous materials using the model with the band gap, respectively. The gray symbols correspond to the MSEs for amorphous materials when the band gap was not included.

The inclusion of band gap information led to only minor improvements for the crystalline materials (orange), indicating that their spectra were already well predicted without the band gap (MSE in Fig. 2a). In contrast, the MSEs for the amorphous materials were significantly improved when the band gap was incorporated (green), compared to the case without the band gap (gray). As a result, the prediction accuracy for the amorphous materials became comparable to that of the crystalline materials (orange).

To analyze it in detail, the predicted spectra without and with a band gap are shown in Fig. 3c and Fig. 3e, respectively. Considering the band gap largely corrected the underestimation of the peak position, namely, the peak shift of the excited state to lower energy. To validate the above model that considered the band gap, we compared it to a model that considered the valence band as an input in addition to the band gap. The valence and conduction bands were the PDOS from −20 to 15 eV, with 0 eV set to the middle of the band gap. The result of this model is shown by purple symbols in Fig. 3a; it did not show more improvement as compared with the MSE for the green symbols, indicating that the band gap is the key factor to predicting the excited state derived from the core hole.

Finally, we considered applying the FNN model to various other materials. Based on the above, it was confirmed that the prediction model can be applied to both crystalline and amorphous materials, even if the model was trained on only crystalline silicon oxides. Here, we applied the trained model to various metal oxides—including Li_2_O (anti-fluorite), MgO (rock salt), Al_2_O_3_ (corundum) and GeO_2_ (α-GeO_2_)—to predict their O–K ELNES/XANES. Note that the local coordination of the metal/oxygen in Li_2_O, MgO, Al_2_O_3_ and GeO_2_ is 4/8, 6/6, 6/4, and 4/2, respectively, whereas the variety of the local structures of silicon and oxygen in the training data are still limited; namely, silicon/oxygen mostly form a 4/2 coordination.

**Fig. 4.**
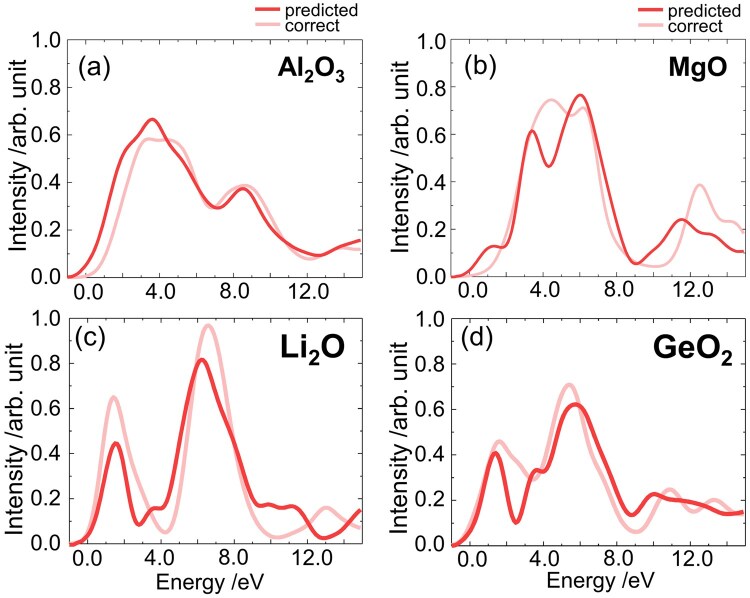
The ELNES/XANES spectra of four metal oxides. Deep and light red lines are the predicted and accurate ELNES/XANES spectra, respectively. (a) Al_2_O_3_, (b) MgO, (c) Li_2_O and (d) GeO_2_. This figure is adapted from reference [148].

Figure 4 shows the predicted and correct O–K edges of Li_2_O, MgO, Al_2_O_3_ and GeO_2_. The model was trained on crystalline silicon oxides, considering both the band gap and valence state. The predicted O–K edge of these oxides was correctly reproduced, even though they had different elements and local structures from the silicon oxides. This successful prediction could provide new insight into the excited state; that is, the impact of the oxygen 1 s core hole on the electronic structure of those oxides is similar to each other. We believe that our model for the ELNES/XANSE in this study was ‘transferable’ and feasible for these oxides [148].

### Acceleration of ELNES/XANES simulation: prediction of ELNES/XANES from text input

To accelerate the simulation of ELNES/XANES, various ML approaches have been proposed. For example, our group recently developed a message-passing neural network capable of accurately predicting site-specific and molecular spectra for the carbon K-edge [154], and as described in the previous section, we have also demonstrated that the PDOS at the ground state is also one of the powerful descriptors to generate the ELNES/XANES spectra [148].

In addition to the atomic coordination, a range of atomic coordinate-dependent descriptors have been widely adopted for spectral prediction due to their strong ability to represent local chemical environments. These include atom-centered symmetry functions [165], the smooth overlap of atomic positions [166], the local many-body tensor representation [167] and the spectral neighbor analysis potential [168].

However, those theoretical calculations and descriptors based on atomic coordinates require explicit atomic configurations. This necessitates structure optimization, which becomes a strong bottleneck for large systems. In such cases, the molecular graph may be known, but accurate atomic coordinates are difficult to obtain, and their optimization is computationally expensive due to the system size. To address this limitation, the use of non-coordinate-based descriptors would be proposed. Here, the utilization of non-atomic coordinate descriptors, such as the SMILES or atomic coordinate-independent molecular graphs, is proposed to surmount this constraint and expedite ELNES/XANES investigations.

SMILES, a linear string notation, succinctly represents molecular structures without requiring atomic coordinate information. This characteristic arises from the inherent lack of direct structural information in its representation, which limits its applicability in molecular dynamics simulations or studies involving amorphous structures. However, its simplicity can reduce the time needed for structure optimization and expedite the acquisition of ELNES/XANES.

To perform this study, we have selected 22 007 molecules without non-bonding structures in the database [155, 169], and the SMILES strings of molecules are obtained from the database directly. These set of molecules are identical to the ones in the previous study in our group, while some of them are removed due to their unbinding structure or inaccessibility of transformation to the SMILES by the RDKit code [170]. To confirm the uniqueness of the SMILES strings for each molecule, we transformed the original SMILES strings into the canonical SMILES strings by the RDKit code. For describing the electronic structure of these molecules, we applied the SMILES to predict carbon K ELNES/XANES.

**Fig. 5.**
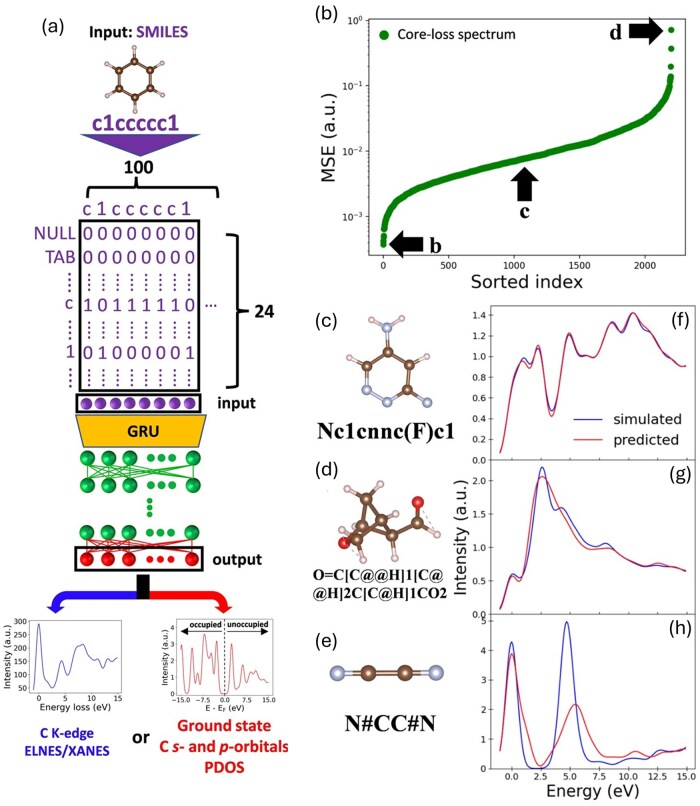
(a) Schematic of the RNN model architecture. The architecture includes an input layer of one-hot presentation of SMILES, an RNN model, and an output layer of carbon s- and p-orbital PDOS (red) and molecular carbon K-edge ELNES/XANES spectrum (blue). (b) Sorted MSE values of the ELNES/XANES spectra prediction. The black arrows show the position of the predictions of the lowest, median, and highest MSE, whose input SMILES strings and molecular structures shown in (c–e). (f–h) The predicted spectra (red curve) and simulated spectra (blue curve) for the molecules shown in (c–e). This figure is adapted from reference [151].

The ML model used in this study is schematically illustrated in Fig. 5a. We employed an RNN [171], consisting of three main components: an input layer, a hidden layer, and an output layer. To enable the processing of SMILES strings in digital format, we converted canonical SMILES into one-hot encoded representations. In this study, each SMILES string was represented as a 100 × 24 one-hot matrix (see Table 1). The first dimension (length 100) corresponds to the maximum allowable length of SMILES strings and defines the number of input nodes in the first hidden layer. The second dimension (length 24) represents the number of unique character types used in the SMILES notation, each encoded as a vector passed into the hidden layer.

**Table 1.** The characters and meaning for transformed SMILES strings in the database.

Character	Meaning	Character	Meaning
NULL	The other characters not on this list	=	Double bond
PAD	Added space when SMILES is shorter than 100	#	Triple bond
(and)	The branch connected with the same element	@	Chiral bond
[and]	The branch connected with other elements	C, F, H, N, O	Element species
1∼5	Ring index or charge value	c, n, o	Element species in aromatic rings
+ and −	Positive and negative charge		

In the hidden layer, we implemented an RNN architecture incorporating a gated recurrent unit (GRU) [172] and a fully connected layer, both implemented using the PyTorch package [173]. The GRU was used to enhance the extraction of sequential information encoded in the SMILES strings and to capture the underlying relationships between the electronic structure and molecular representation. To prevent overfitting, a dropout rate of 0.3 was applied to each layer. For the ELNES/XANES spectrum prediction model, the GRU consisted of three hidden layers with 50-dimensional hidden states. The fully connected network comprised three hidden layers with 4000, 3800 and 1800 nodes, respectively. In this case, the dropout rate was set to 0.05. The ReLU function was used as the activation function throughout the model.

During training, the dataset was split into 80% for training, 10% for validation, and 10% for testing. The MSE was used as the loss function, and the Adam optimizer was employed with a fixed learning rate of 0.000144 to update the model parameters. Early stopping was applied with a patience of 20 epochs to determine convergence and avoid overfitting.

The output layer outputs the molecular carbon K-edge ELNES/XANES spectrum. The range of the ELNES/XANES spectrum is from −1 to 15 eV, where the excitation energy is set at 0 eV. Besides, the increments of these spectra are both 0.1 eV, so the dimension for ELNES/XANES spectrum is 160.

The results of the ELNES/XANES spectrum predicted by the SMILES are also shown in Fig. 5. Figure 5b shows the sorted averaged MSE between the predicted (red line) and simulated (blue line) ELNES/XANES. The black arrows indicate the molecules with the best, median, and worst predictions, whose molecular structures and prediction results are shown in ­Figures 5c–e and 5f–h, respectively. In the best and median prediction performance (Fig. 5f and g), the predicted ELNES/XANES spectral outcomes exhibit a favorable correspondence with the simulated spectra, indicating that most of the ELNES/XANES spectra in the test dataset can be predicted with high accuracy from the corresponding SMILES with our ML model.

As for the worst prediction (Fig. 5h), the intensity of the first main peak in the predicted spectrum is similar to the simulated spectrum, while the second main peak shows an obvious difference. Whereas, the peak position of the second main peak is still similar to the simulated spectrum, indicating that our ML model can still provide reasonable ELNES/XANES spectra prediction even for the worst prediction. Besides, the averaged MSE for test dataset is 0.01 with standard deviation of 0.02, indicating that most of the molecules in the test dataset can still provide acceptable ELNES/XANES.

This clearly indicates that the SMILES, which has information on the molecular structure, can be used as the descriptor to predict the ELNES/XANES. In addition to the ELNES/XANES prediction, we have also demonstrated that the SMILES can be used as the descriptor to predict the local electronic structure, like PDOS [151].

### Beyond conventional analysis: prediction of radial distribution function from ELNES/XANES

Next, we explored the ML application to extract structural information beyond what is conventionally obtained using the ELNES/XANES. As described in the Introduction section, ELNES and XANES reflect electronic transitions from the core level to the unoccupied PDOS in the conduction bands. Therefore, they contain the information about the PDOS in the conduction band at the excited state. However, when one aims to extract information related to the local atomic coordinates, such as coordination numbers or bond length, it is necessary to analyze the extended energy region beyond the ELNES/XANES, which corresponds to the extended energy loss fine structure (EXELFS) or extended X-ray absorption fine structure (EXAFS) [174]. By extracting the oscillations in the EXELFS/EXAFS region and applying Fourier transformation, one can obtain the RDF, where the peak positions correlate with bond lengths and the intensities are related to coordination numbers.

In the experimental observation, EXELFS/EXAFS measurements require the acquisition of spectral data extending up to ∼1000 eV beyond the spectral threshold to avoid overlapping with other absorption edges in this energy range; higher-energy edges, usually K-edge, are typically used for the EXELFS/EXAFS measurements. While such measurements are widely performed at synchrotron facilities, they are less common in electron microscopes due to the limited energy limitation, typically up to 3 keV, of conventional spectrometers. Although new spectrometer designs have enabled measurements up to ∼35 keV, expanding the feasibility of EXELFS analysis using EELS, such high-energy measurements still face challenges, such as signal attenuation, longer acquisition times and increased risk of beam-induced damage.

To address these limitations, we attempted to directly extract the RDF from ELNES/XANES region using the ML. Specifically, we constructed a spectral database of O–K edge of silicon oxides comprising pairs of ELNES/XANES profiles and their corresponding RDFs and developed a prediction model based on an FNN to infer RDFs directly from the spectral data.

**Fig. 6.**
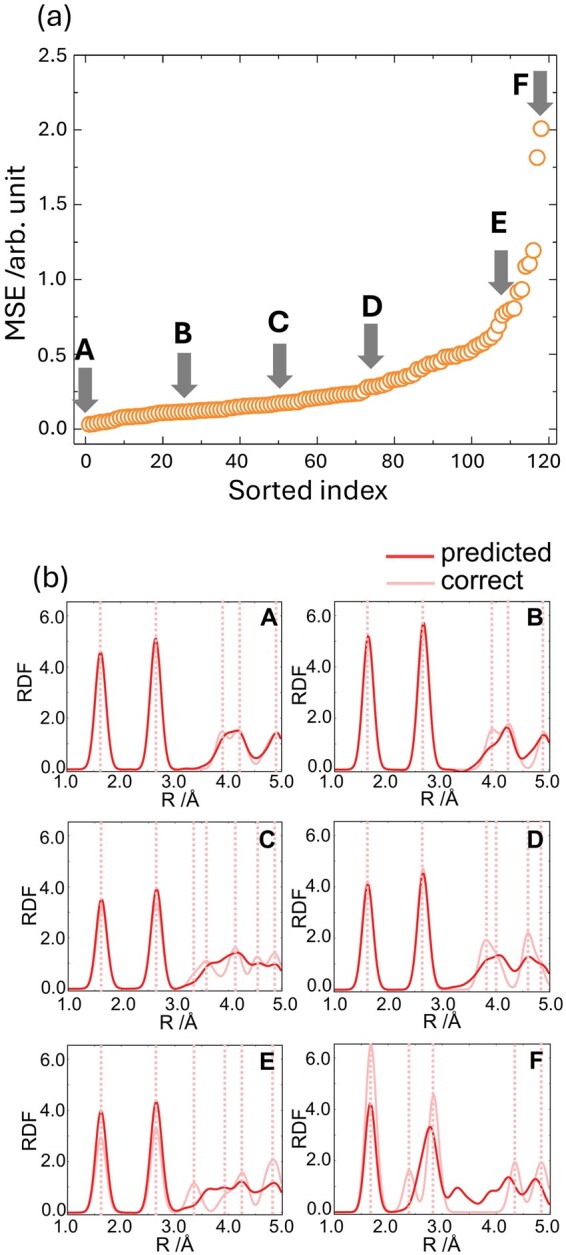
(a) Sorted MSEs are scattered by orange open circles. (b) Predicted and correct RDFs. Solid deep-red and light-red lines are the predicted and correct RDFs, respectively. Dotted light-red lines represent the peak positions of the correct RDFs. Bold alphabet characters **A** to **F** correspond to those in (a). This figure is adapted from reference [149].

The performance of the FNN model was evaluated using the MSE on the test dataset. Figure 6a presents the MSEs of the test samples, where the *x*-axis indicates the sample index sorted in ascending order of MSE, and the *y*-axis shows the corresponding MSE values. The MSEs increase gradually up to approximately sample index 110 (around point E), after which they rise more steeply. Overall, more than 90% of the test samples exhibit MSEs below 0.5, while the remaining cases show significantly larger prediction errors.

The predicted RDFs corresponding to points A to F are shown in Fig. 6b. The predicted and correct RDFs are shown in deep red and light red, respectively. All RDF profiles exhibit two sharp peaks between 1 and 3 Å, followed by broader features at distances beyond 3 Å. These peaks correspond to bond lengths and coordination numbers around the oxygen atoms at the excited state. With respect to the labels A–D, their predicted RDFs from 1 to 3 Å agree well with their correct RDFs. However, the predicted RDFs of E and F have relatively larger errors even with RDFs of 1 to 3 Å. In the much longer range (3–5 Å), point A still shows good correspondence at the peak intensity and position, whereas the peak positions are in good agreement with each other in Samples B to D, even in the range of 3–5 Å, while the intensities have some deviations. The predicted error in the longer range looks large at points E and F. We found that the large error was caused by the crystal structure corresponding to the data points over point E, which has considerably unique atomic structures, a rare condition in existing datasets.

In general, ELNES/XANES provides information about local chemical bonding, while EXAFS is known to capture middle-range structural features such as first- and second-neighbor coordination shells. Consequently, ELNES/XANES and EXELFS/EXAFS are often used for complementary purposes in bonding and coordination analysis. The successful prediction of RDFs from ELNES/XANES using the FNNs suggests that ELNES/XANES spectra intrinsically contain sufficient middle-range structural information, which can be extracted through appropriate learning models. These results demonstrate that ELNES/XANES is not limited to probing only local electronic structure but can also reflect the coordination information, such as bond distances and coordination numbers, beyond the nearest neighbor.

**Fig. 7.**
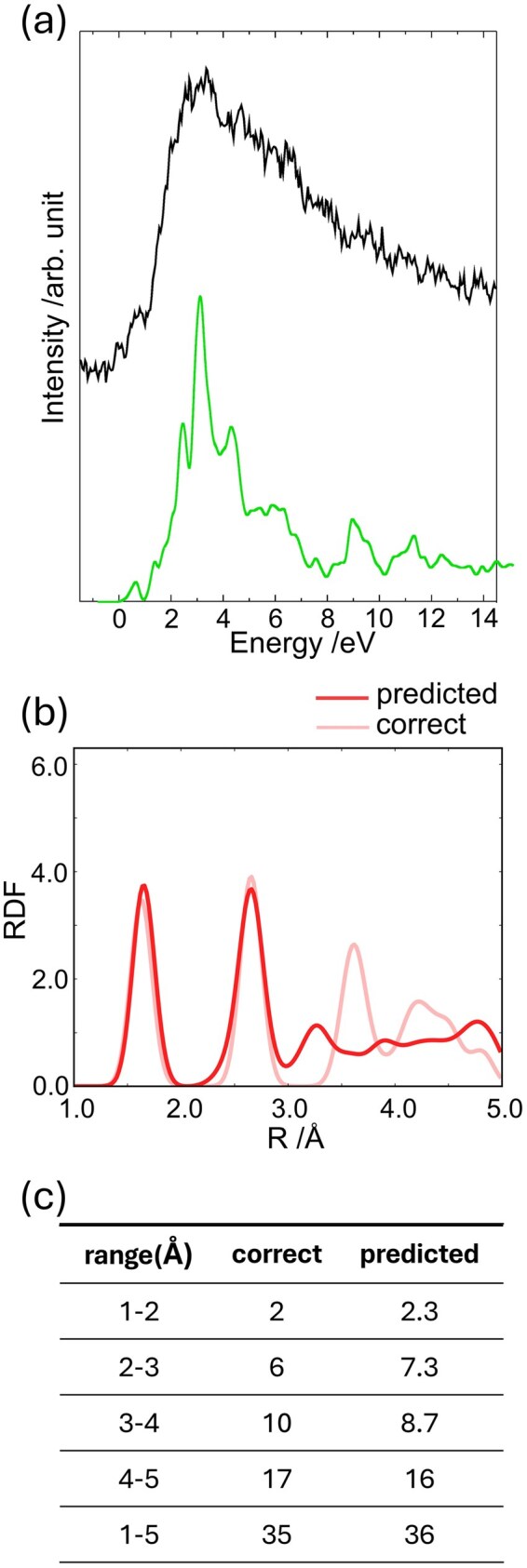
(a) Experimental O–K edge ELNES of α-quartz. The spectral onset was set to 0 eV. (b) Predicted and correct RDFs of α-quartz. (c) Predicted and correct coordination numbers estimated from the corresponding RDF. This figure is adapted from reference [149].

Finally, we applied the constructed FNN model to an experimental O–K edge spectrum of SiO_2_, α-quartz. The spectrum was acquired in our previous studies [88, 89]. The experimental O–K edge spectrum and the RDF predicted from it are shown in Fig. 7a and b, respectively. The predicted RDF (deep red) agrees well with the reference RDF (light red). Figure 7c presents the estimated coordination numbers within defined radial ranges.

Although the experimental spectrum contains significant noise, the predicted RDF in the short-range region (1–3 Å) was accurately reproduced, with an estimated coordination number error of ∼0.3. This result demonstrates that the FNN model, trained solely on simulated data, is applicable to the determination of local atomic structures from such experimental spectra. In contrast, the predicted RDF in the middle-range region (3–5 Å) showed relatively larger deviations, particularly in peak positions, and the estimated coordination number error was around 1 (Fig. 7c).

These results indicate that XANES/ELNES spectra, despite being limited to the near-edge region, contain structural information not only about the local bonding environment but also about coordination environments within a few sub-nano meters. Such information can be extracted through ML, enabling structural analysis beyond the conventional scope of near-edge spectroscopy.

### Beyond conventional analysis: quantification of multiple properties from ELNES/XANES

Here, I would introduce an approach in which material properties are directly predicted from ELNES/XANES spectra via ML approach. While ELNES/XANES spectra offer insights, like atomic and electronic structures, the correlations between those spectral features and material properties are not fully understood. In particular, the correlation between ELNES/XANES and extensive properties, such as molecular weight, needs further exploration. If such relationships can be established through ML, it would enable the property predictions, such as optical characteristics, electrical conductivity, density and stability, with high sensitivity, spatial resolution and temporal resolution, leveraging the advantages of ELNES/XANES. Moreover, once this link is established, material property prediction and design could also be performed based on calculated ELNES/XANES spectra.

Here, a ML framework to directly and quantitatively predict the hidden information on material properties from ELNES/XANES spectra is introduced. Specifically, we utilized structural information of organic molecules from existing molecular databases [155, 169], simulated their carbon K-edge ELNES/XANES spectra for a total of 22 155 molecules, and used these spectra as input to a neural network model to quantify molecular properties. In this approach, the ELNES/XANES spectra functioned as descriptors to represent the materials and enabled quantitative predictions of their properties.

Although accurate predictions of molecular properties have been reported using various ML models, such as SchNet [119], deep neural networks (DNNs) [118], graph convolutional multitask models [175], deep tensor neural networks (DTNNs) [176] and message passing neural networks (MPNN) [177], these approaches rely heavily on chemically dedicated descriptors. For example, MPNNs require predefined chemical features such as donor/acceptor groups and hybridization states; DTNNs and SchNet models employ a large number of learned parameters; and DNNs and multitask learning approaches depend on detailed atomic coordination information. As a result, atomic- or electronic-level descriptors are essential for these prediction models, while such inputs are often unavailable or difficult to measure experimentally.

In contrast, the descriptor used in the present study, that is, core-loss spectrum, offers a distinct advantage: it is directly measurable using experimental techniques such as EELS and XAS. Furthermore, modern instrumentation enables such measurements with high sensitivity, spatial resolution and temporal resolution. Therefore, the approach proposed here has strong potential for materials development, as it can reveal where, when, and how specific material properties emerge, using spectra that are experimentally accessible.

**Fig. 8.**
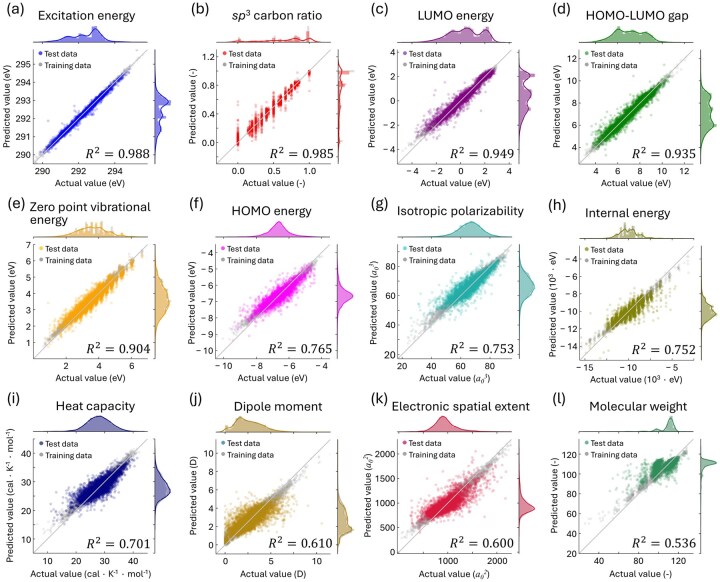
Parity plots comparing the actual values to the predicted values for the 12 properties investigated: (a) excitation energy, (b) *sp*^3^ carbon ratio, (c) LUMO energy, (d) HOMO–LUMO gap, (e) zero-point vibrational energy, (f) HOMO energy, (g) isotropic polarizability, (h) internal energy, (i) heat capacity, (j) dipole moment, (k) electronic spatial extent and (l) molecular weight. The *a*_0_ is the Bohr radius. The *R*^2^ value given at the bottom of each figure represents the coefficient of determination, which ranges from 0 to 1. The results are displayed in order of their accuracy (*R*^2^); namely, the accuracy decreases from (a) to (l). The horizontal axes show the actual values, and the vertical axes show the predicted values. The colored and gray circles represent test and training data, respectively. A circle present on the gray diagonal line indicates that the predicted value is exactly equal to the actual value. The distribution of the training and test data are shown adjacent to their corresponding axes. This figure is adapted from reference [150].

Based on this background, we constructed ML models to quantify 12 molecular properties from the carbon K-edge (C-K edge) spectrum of each molecule. The target properties included excitation energy, *sp*³ carbon ratio, LUMO energy, HOMO–LUMO gap, zero-point vibrational energy, HOMO energy, isotropic polarizability, internal energy, heat capacity, dipole moment, electronic spatial extent and molecular weight. The prediction results are shown in Fig. 8a–l, ordered by descending prediction accuracy, from the highest in Fig. 8a to the lowest in Fig. 8l. For several properties, including excitation energy, *sp*³ carbon ratio, LUMO energy, and HOMO–LUMO gap, the coefficient of determination (*R*^2^) exceeds 0.93, indicating high prediction performance. Most properties were predicted with reasonable accuracy (*R*^2^ > 0.7). In contrast, the prediction of molecular weight showed relatively low accuracy, with an *R*^2^ value of ∼0.54.

Typically, the carbon K-edge spectrum reflects the p-type PDOS of unoccupied orbitals in carbon atoms. However, as demonstrated in Fig. 8, the results suggest that the ELNES/XANES can serve as a powerful descriptor for predicting a wide range of molecular properties, including some that are inherently related to the electronic structure of occupied orbitals in the ground state. This type of ‘breakthrough’ prediction from unoccupied to occupied electronic states can be understood by the following considerations. First, the formation of unoccupied orbitals is fundamentally linked to that of occupied orbitals. For example, σ- or π-bonding orbitals in the occupied state are typically accompanied by corresponding σ*- or π*-antibonding orbitals in the unoccupied state. In other words, the unoccupied state electronic structure inherently contains information about the occupied states.

Contrary to the excitation energy, the *sp*^3^ carbon ratio, and the HOMO–LUMO gap, the *R*^2^ value for the molecular weight, which was 0.536, is quite worse than others (Fig. 8l). This is reasonable because the molecular weight is one of the extensive properties. An extensive property is a size-dependent property, and it can be expected that such extensive properties do not directly correlate to the electronic structure.

We have already revealed the way to improve the molecular weight prediction [150]. To improve the prediction accuracy, the relative compositions, in particular, the ratios of nitrogen, oxygen and fluorine to carbon, were focused, because the compositional information can be easily accessed experimentally using other spectroscopic methods. Furthermore, the possible values for the output can be limited by including this additional information in the model. Three additional data, the ratio of nitrogen, oxygen and fluorine atoms to carbon atoms in a molecule, were used as input data in addition to the 240-dimensional C-K edge input data. In total, 243-dimensional input data were used. Then, we have confirmed that the molecular weight prediction obtained using the updated model achieved [150].

Furthermore, in addition to the individual predictions, we have also performed the simultaneous quantification of all properties from the single spectrum [150]. The prediction accuracy does not get worse largely even in the simultaneous predictions. Namely, C-K ELNES/XANES is quite powerful descriptor to predict those molecular properties simultaneously. The advantage of the descriptor proposed in this study is that it is experimentally observable. Our study proposed machine-learning-based property measurements using the core-loss spectra, which can benefit high sensitivity, high spatial resolution, and high temporal resolution from modern EELS and XAS instrumentation.

Our study demonstrates the potential of machine-learning-based property estimation using ELNES/XANES, which are experimentally accessible and can benefit from the high sensitivity, spatial resolution, and temporal resolution offered by modern EELS and XAS instrumentation.

### Interpretation of the prediction model using sensitivity analysis

Thus far, we have demonstrated that ML is effective for data-driven analysis of ELNES/XANES. However, it is known that the ML is usually ‘black box’. That is, even when high prediction accuracy can be achieved, the internal decision-making process of the model often remains opaque. In particular, the models used for the property prediction typically exhibit high expressive power and strong nonlinearity, making it difficult to interpret the learned relationships in a physically meaningful way. To address this issue, we conducted a sensitivity analysis of the constructed FNN model [178]. By applying the sensitivity analysis to the prediction model for the molecular property introduced in ‘Beyond conventional analysis: quantification of multiple properties from ELNES/XANES’ section, an insight into how the model makes predictions would be revealed.

**Fig. 9.**
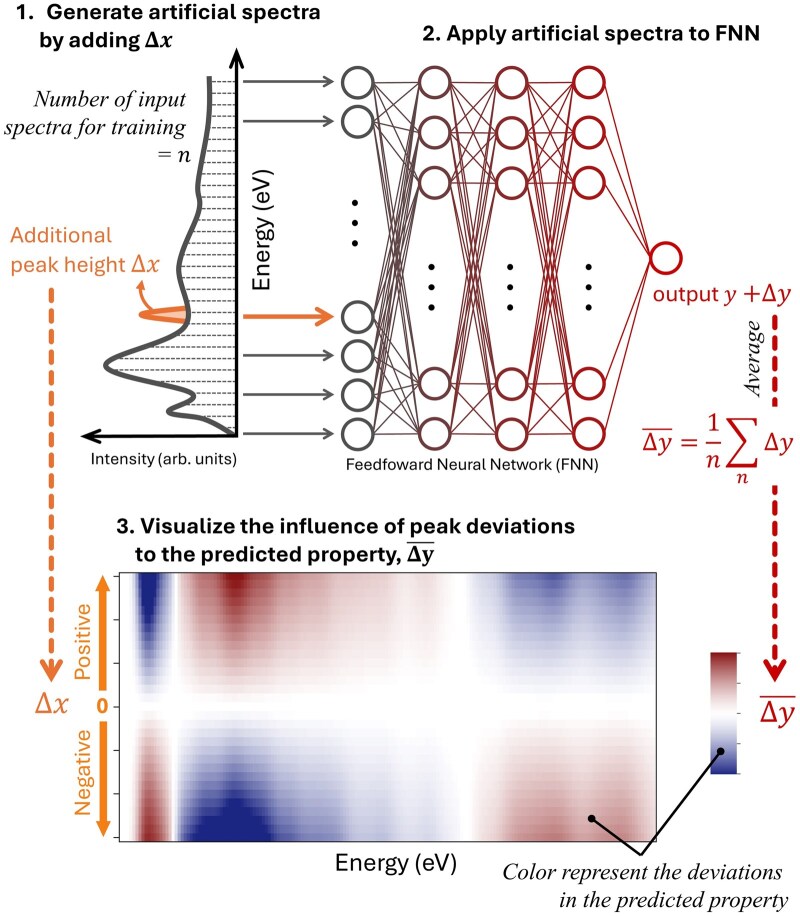
Schematic illustration of the sensitivity analysis. (Top-left) First, an artificial spectrum was generated by adding additional peak, Δx, at specific energy. (Top-right) Apply the artificial spectrum to FNN and check the deviation of the predicted value, Δy. The same process was performed with different Δx, and continued to all energies (node) of spectral dataset for the training data, and obtain average of Δy, $\overset{-}{\Delta y}$. (Bottom) Obtained $\overset{-}{\Delta y}$ at the respective energy with different Δx were plotted with color map. This figure visualizes the resultant deviation in the property values (red and blue) induced by the increase/decrease of the additional peak (vertical axis) at various energies (horizontal axis). This figure is adapted from reference [150].

This analysis is schematically illustrated in Fig. 9. Artificial input spectra were generated by superimposing Gaussian-­shaped peaks (orange *Δx* in Fig. 9) at various energy positions onto the original training spectra. These modified spectra were then input into the trained FNN. Both positive and negative peak perturbations were applied in order to evaluate the influence of increasing or decreasing spectral intensity on the predicted excitation energy. The average effect of these peak modifications on the predicted property, denoted as ($\overset{-}{\Delta y}$), was computed and mapped as a function of energy and *Δx*, as shown in Fig. 9.

**Fig. 10.**
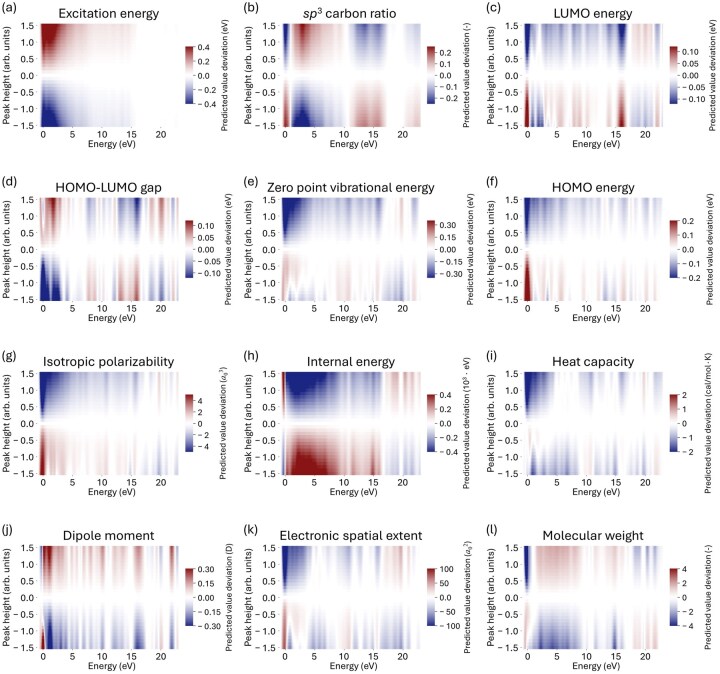
Sensitivity analyses with color plots showing deviations in the predicted values of the 12 properties investigated after perturbation of an artificial Gaussian peak with an energy at the height of the deviation: (a) excitation energy, (b) *sp*^3^ carbon ratio, (c) LUMO energy, (d) HOMO–LUMO gap, (e) zero-point vibrational energy, (f) HOMO energy, (g) isotropic polarizability, (h) internal energy, (i) heat capacity, (j) dipole moment, (k) electronic spatial extent and (l) molecular weight. This figure is adapted from reference [150].

The results of the sensitivity analysis for all predicted properties are summarized in Fig. 10. As an example, Fig. 10a shows the changes in predicted excitation energy induced by artificial peak perturbations. The vertical axis represents the magnitude of the added peak (positive or negative), while the horizontal axis indicates the energy position of the perturbation. Red and blue colors correspond to increases and decreases in the predicted property, that is, excitation energy, respectively. This visualization allows us to evaluate how variations in spectral intensity at different energy regions affect the predicted property. Through this sensitivity analysis, it can be revealed that the perturbations near the threshold region (0–2 eV) significantly affect the predicted excitation energy. Specifically, an increase in spectral intensity in this region tends to raise the predicted excitation energy (red), whereas a decrease results in a lower predicted value (blue). These results indicate that spectral features near the threshold are closely correlated with excitation energy and that the FNN model successfully learned this relationship during training.

A similar trend is observed for the sp³ carbon ratio, as shown in Fig. 10b. In core-loss spectroscopy, it is well known that the first sharp peak in the carbon K-edge corresponds to transitions to π* orbitals, while higher-energy features are associated with σ* orbitals. Accordingly, a prominent π* peak near the threshold implies a greater contribution from sp^2^-hybridized carbon, leading to a lower sp³ carbon ratio. In contrast, the presence of σ* features at higher energies is indicative of a higher sp³ content. The sensitivity analysis in Fig. 10b shows that the model correctly captured these relationships: perturbations near the π* region reduce the predicted sp³ ratio, while those in the σ* region increase it. These findings demonstrate that the FNN model was able to extract chemically meaningful features from the spectra, relationships that typically require expert interpretation, and quantify the sp³ carbon ratio across diverse molecular systems.

In the same manner, sensitivity analysis enabled us to identify which features in the C-K edge spectrum, in particular, increases or decreases in individual peaks, correlate with changes in particular material properties. However, it is important to note that the present sensitivity analysis considers only single-peak variations. In reality, changes in electronic structure that influence material properties can be expected to involve not just one peak, but collective variations across multiple spectral features. Although our current results do not capture such complex relationships, the use of similar sensitivity analysis approaches may provide a path forward for identifying correlations between spectral shape variations and material properties in more comprehensive ways.

### Conclusion and outlook

In this manuscript, I reviewed recent developments of data-driven approaches for ELNES/XANES by the author’s group, in particular predictions of ELNES/XANES, radial distribution function and multiple properties.

First, I introduced a high-throughput prediction for ELNES/XANES based on the ground-state PDOS, which can be quickly calculated using the primitive cell. We demonstrated that O–K ELNES/XANES spectra of crystalline Si–O compounds can be accurately predicted from ground-state O–p PDOS. When the prediction model trained using the crystalline Si–O compounds was applied to the amorphous Si–O compounds, the prediction accuracy decreased significantly. This result led to the finding that the core-hole effects of the O–K edge in the Si–O system are more pronounced in amorphous structures than in the crystalline system. I also showed that the incorporation of the band gap as an additional input improves the prediction accuracy for amorphous materials. I further showed that the trained model could be extended to other ionic oxides, demonstrating its transferability across materials with similar chemical characteristics.

In addition, I introduced the method to predict the C–K edge spectra directly from SMILES representations using an RNN. This approach enables the prediction of core-loss spectra without requiring atomic coordinates and any simulations, which is particularly beneficial for large molecules where structure optimization is computationally demanding or infeasible.

I also presented that ELNES/XANES spectra can serve as effective descriptors for structural prediction. Specifically, a model that predicts the RDF around oxygen atoms from O–K edge spectra of Si–O compounds was shown. Furthermore, by applying the trained model to experimental O–K edge spectra of α-quartz, I showed that RDFs can be accurately predicted up to ∼3 Å, even when using the experimental spectrum as input.

Moreover, I presented an application of ML in which the C–K edge spectra were used to quantify 12 molecular properties, such as excitation energy, HOMO–LUMO gap and molecular weight. While intensive properties were predicted with high accuracy (e.g. *R*^2^ > 0.9 for the excitation energy), extensive properties such as molecular weight showed lower performance (*R*^2^ ∼ 0.54). To address this limitation, we developed an improved model that successfully enhanced prediction accuracy for molecular weight.

Finally, I also touched on an approach to address the issue that the ML-based modeling has a black-box nature of the prediction process. We used sensitivity analysis on the molecular property prediction model. This approach allowed us to visualize the relationship between changes in spectral features (e.g. intensity at specific peaks) and changes in predicted property values, providing insight into how the model interprets spectral input.

Importantly, the proposed ML approach is not limited to ELNES/XANES spectra. Since it does not rely on spectroscopy-specific assumptions, it can, in principle, be extended to other types of spectral or experimental data, such as diffraction patterns or emission spectra.

Furthermore, the effect of experimental noise, which is inevitably present in measured spectra, is an important consideration for the practical application of ML methods. In this review, all the models discussed were constructed using simulated spectra, which are inherently noise-free. Nevertheless, as demonstrated in Fig. 7 (‘Beyond conventional analysis: prediction of radial distribution function from ELNES/XANES’ Section), the model successfully predicted the radial distribution function up to 3 Å even when applied to experimental spectra, suggesting that models trained on simulated data can, to some extent, be directly transferred to experimental datasets.

In our previous studies [145, 146, 152, 153], we systematically examined the effect of noise on prediction accuracy. These studies revealed that experimental noise is not a major obstacle for most tasks. In fact, the most accurate models can often be obtained by introducing simulated noise levels comparable to those in the target experimental data [153]. By contrast, the limiting factor is not noise itself but rather the energy resolution. Excessively noisy spectra that obscure fine structural features are unusable, but spectra with sufficiently high-energy resolution retain the essential fine features related to local structure and chemical bonding, which are crucial for ML applications [152].

Another critical issue concerns the use of experimental spectra as direct input for ML. Constructing accurate and robust models requires not only a large quantity of experimental data but also high-quality metadata. While moderate noise levels in the spectra are manageable, errors or ambiguities in metadata, such as incorrect sample identification, damaged spectra or measurements limited to specific orientations, would pose more serious challenges. Reliable annotation of such conditions (e.g. ‘reduced by damage’ or ‘measured along <100> direction’) would enhance the usability of experimental data for ML.

Ultimately, the establishment of sufficiently large, well-curated and reliable experimental spectral databases, with both high-quality spectra and accurate metadata, will be essential for building accurate ML models for the experimental data.

Recently, large-scale spectral databases containing sub-million-level calculated spectra have been constructed [155, 179, 180]. Combining such databases with the present method could facilitate highly versatile and accurate spectral interpretation.

For instance, experimental databases are summarized on the following web pages:

International XAFS DB Portal: https://ixdb.jxafs.org/

xrayabsorption.org: https://xafs.xrayabsorption.org/databases.html

The Japanese XAFS community has made significant contributions to the construction of experimental databases for XANES and EXAFS, as shown in the following:

https://doi.org/10.48505/nims.1447

https://pfxafs.kek.jp/xafsdata/list.php

https://www.jxafs.org/xafs-database/

Other XAFS communities have also constructed databases, such as:

Italy: https://lisa.iom.cnr.it/xasdb/

US: https://xaslib.xrayabsorption.org/elem/

China: https://xasdb.ihep.ac.cn/

France: https://www.esrf.fr/UsersAndScience/Experiments/XNP/ID21

France: https://www.sshade.eu/doi/10.26302/SSHADE/FAME

Canada: https://xasdb.lightsource.ca/

Similarly, EELS databases are available at:

https://eelsdb.eu/

https://eels.info/atlas

Furthermore, databases of simulated spectra using the multiple scattering method FEFF are well summarized on the following web page:

https://next-gen.materialsproject.org/

Although the following database is still limited the simulated electronic structure data, great efforts are making for collecting experimental data:

https://nomad-lab.eu/nomad-lab/

Large collections of simulated spectra have also been published in database journals [155, 179–181]. In addition to spectral datasets, electron microscopy image data have been reported in such journals as well [182–184]. Finally, software tools for spectral data analysis are also important, and these have been comprehensively summarized in other manuscripts [125].

Furthermore, the rapid advancement of ML models, particularly large language models (LLMs) such as ChatGPT and Gemini, has opened new possibilities in spectral and microscopic analysis. For example, atomic-resolution images containing lattice defects can be interpreted through prompt-based interaction with LLMs. Similarly, some spectra uploaded as CSV files can be analyzed and interpreted directly using LLM. While the accuracy of such AI-based interpretations remains a subject of debate, it is important to recognize that their performance is improving dramatically and rapidly with each passing day.

In materials science, generative AI models based on graph neural networks combined with diffusion models are also being developed [96, 98, 99, 185]. An inverse design of materials for the target properties would be available using such modern architecture. In addition, prompt-driven ‘vibe coding’ using LLMs is now being applied to automate experimental workflows, including device control, measurement, and data analysis [186–190]. Our group is actively exploring such technologies to advance next-generation spectral analysis, which will be reported elsewhere in the near future.

In summary, the data-driven approaches presented in this review offer a transformative pathway for spectral analysis, materials characterization and local property prediction. By bridging physics-based insights with the scalability of ML, these methods enable not only faster and more accurate interpretation of complex spectral data but also open new frontiers in materials discovery. As the landscape of AI continues to evolve, encompassing large language models, generative architectures and automated experimental design, our ability to design, understand and optimize the target materials will be fundamentally redefined. The convergence of advanced ML and spectroscopy marks a pivotal shift toward a future of autonomous, interpretable and data-centric materials research.
